# Cepharanthine Blocks Presentation of Thyroid and Islet Peptides in a Novel Humanized Autoimmune Diabetes and Thyroiditis Mouse Model

**DOI:** 10.3389/fimmu.2021.796552

**Published:** 2021-12-20

**Authors:** Cheuk Wun Li, Roman Osman, Francesca Menconi, Larissa C. Faustino, Kookjoo Kim, Oliver B. Clarke, Hanxi Hou, Yaron Tomer

**Affiliations:** ^1^ The Fleischer Institute for Diabetes and Metabolism, Department of Medicine, Albert Einstein College of Medicine, New York, NY, United States; ^2^ Department of Pharmacological Sciences, Icahn School of Medicine at Mount Sinai, New York, NY, United States; ^3^ Endocrinology Unit, University of Pisa, Pisa, Italy; ^4^ Department of Anesthesiology, Columbia University, New York, NY, United States; ^5^ Department of Physiology, Columbia University, New York, NY, United States

**Keywords:** autoimmune thyroiditis, type 1 diabetes, autoimmune polyglandular syndrome, cepharanthine, immune therapy

## Abstract

Autoimmune polyglandular syndrome type 3 variant (APS3v) refers to an autoimmune condition in which both type 1 diabetes (T1D) and autoimmune thyroiditis (AITD) develop in the same individual. HLA-DR3 confers the strongest susceptibility to APS3v. Previously we reported a unique amino acid signature pocket that predisposes to APS3v. We found that this pocket is flexible and can trigger APS3v by presenting both thyroid (Tg.1571, TPO.758) and islet (GAD.492) peptides to induce autoimmune response. We hypothesized that blocking the specific APS3v-HLA-DR3 pocket from presenting thyroid/islet antigens can block the autoimmune response in APS3v. To test this hypothesis we performed a virtual screen of small molecules blocking APS3v-HLA-DR3, and identified 11 small molecules hits that were predicted to block APS3v-HLA-DR3. Using the baculovirus-produced recombinant APS3v-HLA-DR3 protein we tested the 11 small molecules in an *in vitro* binding assay. We validated 4 small molecule hits, S9, S5, S53 and S15, that could block the APS3v-HLA-DR3 pocket *in vitro*. We then developed a novel humanized APS3v mouse model induced by co-immunizing a peptide mix of Tg.1571, TPO.758 and GAD.492. The immunized mice developed strong T-cell and antibody responses to the thyroid/islet peptides, as well as mouse thyroglobulin. In addition, the mice showed significantly lower free T4 levels compared to controls. Using the APS3v mouse model, we showed that one of the 4 small molecules, Cepharanthine (S53), blocked T-cell activation by thyroid/islet peptides *ex vivo* and *in vivo*. These findings suggested Cepharanthine may have a therapeutic potential in APS3v patients carrying the specific APS3v-HLA-DR3 pocket.

## 1 Introduction

Type 1 diabetes (T1D) and autoimmune thyroid diseases (AITD) frequently develop together in the same individual, a condition referred to as autoimmune polyglandular syndrome type 3 variant (APS3v) (1–5). The hallmarks of both AITD and T1D are the infiltration of the target glands, pancreatic islets in T1D and thyroid in AITD, by autoreactive lymphocytes and production of antibodies against self-antigens, leading to target organ damage and clinical symptoms (4, 6).

Previous studies by our group and others have shown a strong genetic effect on the development of APS3v (5), with HLA-DR3 showing the strongest association (7–11). Mechanistically we have shown that HLA-DR3 conferred a strong risk for T1D+AITD (APS3v) by facilitating the presentation of islet and thyroid antigens within a flexible HLA-DR3 peptide binding pocket (10, 12). We identified 4 islet and thyroid peptides (Tg.1571, GAD.492, TPO.758 and TPO.338) that elicited strong T-cell responses in humanized DR3 mice carrying the flexible APS3v-HLA-DR3 peptide binding pocket (12). Therefore, we hypothesized that blocking the flexible HLA-DR3 pocket can be used as a new targeted therapeutic approach to treat and/or prevent T1D+AITD (APS3v).

**Figure 1:**
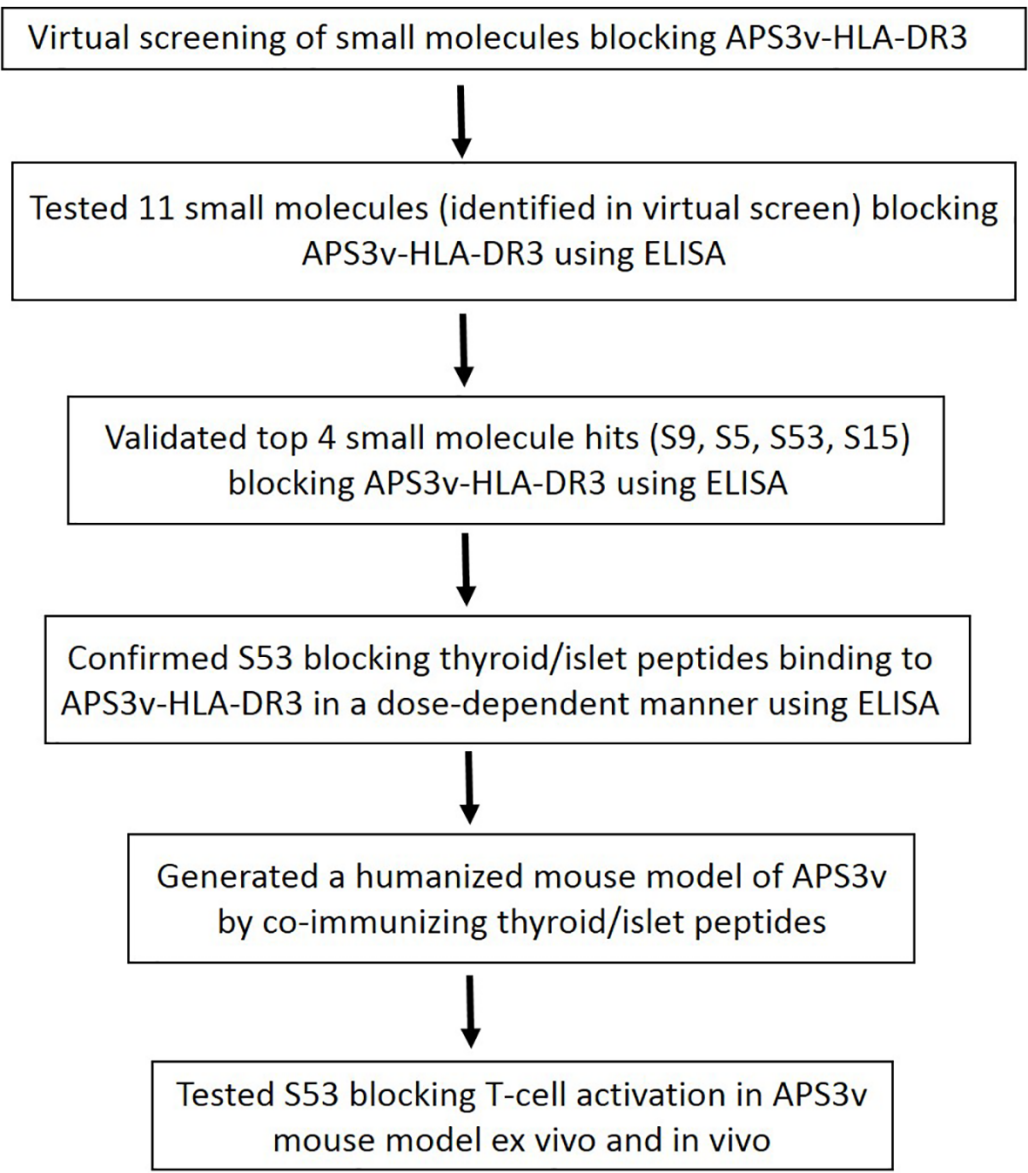
A flow chart showing the approach we took to identify Cepharanthine (S53) as a compound that can block HLA-DR3 in APS3v. First we performed a virtual screen which identified 11 compounds. This was followed by ELISA testing which we validated 4 top hit compounds out of the 11 predicted by the virtual screen (S9, S5, S53 and S15). We then generated a humanized mouse model of APS3v and tested whether S53 can block T-cell activation in the APS3v mouse model both *ex vivo* and *in vivo*. S53 was selected for further testing because it is the only compound out of the 4 validated small molecules that has been used in humans before.

Here we report the identification of a compound, Cepharanthine, that can specifically block the flexible APS3v-HLA-DR3 pocket. Cepharanthine blocked HLA-DR3 both *in vitro* and *in vivo* in a novel APS3v mouse model we have developed. **Figure 1** shows the methodological approach of our study. Our results suggest that Cepharanthine may be effective in preventing/treating disease in patients with T1D+AITD (APS3v).

## 2 Materials and Methods

### 2.1 Production of Recombinant APS3v-HLA-DR3

APS3v-HLA-DR3 protein was produced using the baculovirus system as we have previously described using the Life Technologies Baculovirus protein production custom services (Carlsbad, CA, USA) (12). The sequences of alpha and beta chain of APS3v-HLA-DR3 protein are shown in **Supplementary Table 1**.

### 2.2 Peptide Synthesis

Peptides were custom-synthesized by Genscript (Piscataway, NJ). The following peptides were used in the study: (1) the thyroglobulin (Tg) peptide Tg.1571; (2) the glutamic acid decarboxylase 65 (GAD65) peptide GAD.492; (3) and the thyroid peroxidase (TPO) peptide TPO.758 (12). The apopeptide (APO) that was previously shown by us (13) and others (14) to be the best binder to the HLA-DR3 pocket was used in our initial small molecule screening. The sequences of the peptides are shown in **Table 1**.

**Table 1:** Sequence of peptides used in ELISA screening or immunizations.

Peptide	Sequence
Tg.1571	EKVPESKVIFDANAPVAVRSKVPDSEF
GAD.492	REGYEMVFDGKPQHTNVCF
TPO.758	ESGRRVLVYSCRHGYELQG
APO	IPDNLFLKSDGRIKYTLNK

### 2.3 Virtual Screening of Small Molecules Blocking APS3v-HLA-DR3

#### 2.3.1 Construction of the APS3v-HLA-DR3 Model

The sequence of APS3v-HLA-DR3 is very similar to that of HLA-DRβ1-Arg74 with only three substitutions (all in the β-subunit N37Y, F47Y, V86G). Although they are proximal to the binding groove, from inspection of the structure of HLA-DR3 it is clear that they are not in direct interaction with the peptides. Nevertheless, we wanted to perform the virtual screen on a structure that contains the substitutions. We constructed the APS3v-HLA-DR3 by introducing the three substitutions in the DR3 structure from our previous simulations with Tg.1571 (13). The structure was placed in a periodic box with neutralizing ions and waters. The system was minimized, heated and equilibrated as described in previous work (12). We collected a trajectory from a simulation of 500 ns. The peptide was removed and the structures from the trajectory were analyzed by 2D-RMSD. A representative structure from the largest cluster was used in the virtual screening.

#### 2.3.2 Virtual Screening of the APS3v-HLA-DR3

Virtual screening was conducted with the Schrodinger software using the eMol library available in the Schrodinger suit of programs (discontinued). The same library was used before in virtual screening of the HLA-DRβ1-Arg74 (15). The top 100 poses were extracted from the docking using Glide (16).These were used to conduct an Induced Fit (IF) docking and the results were ordered by the IF-score.

### 2.4 *In Vitro* Screening of Small Molecules Blocking APS3v-HLA-DR3

The virtual screen of 150,000 compounds yielded 11 top scoring small molecules, predicted to block the flexible APS3v-HLA-DR3 pocket, and they were selected for further confirmatory testing. These 11 compounds were purchased from Chembridge (San Diego, CA) or from Microsource Discovery Systems (Gaylordsville, CT). The compounds were dissolved in 100% DMSO (American Type Culture Collection, Manassas, VA) and tested for their ability to block peptide binding to recombinant APS3v-HLA-DR3 as described previously (12, 15). Briefly, 0.012 mg/ml of recombinant APS3v-HLA-DR3 was incubated with 10 µM biotinylated peptides [APO, Tg.1571, GAD.492, or TPO.758 (Genscript) as indicated in Results], together with 0.4 mM of the small molecules, for 48 h at 37 °C in a binding buffer [0.1% BSA/PBS with 0.05% Triton (PBST), Sigma-Aldrich]. On the day before the immunoassay was performed, a 96-well DELFIA yellow plate (PerkinElmer Life Science) was coated overnight with 20 µg/ml of L243 antibody [Hybridoma was purchased from ATCC, catalogue number HB-55, and IgG was purified by QED Bioscience (San Diego, CA)] in bicarbonate buffer, pH 9.6 (Sigma-Aldrich). L243 is a monoclonal antibody that specifically recognizes the DRα chain of HLA-DR when it is properly folded and complexed with the β chain. The plate was then washed with DELFIA wash buffer (diluted 1:25 from DELFIA wash concentrate, PerkinElmer) to wash off the excess L243 antibody. Blocking was performed using 2.5% BSA in PBS at room temperature for 1 h. After washing 4 times, 100 µl of the pre-incubated mix (containing recombinant APS3v-HLA-DR3 protein, APO, and small molecules) were added onto the plate and shaken at slow speed for 2 h at room temperature. After washing 4 times, DELFIA Europium-labeled streptavidin (PerkinElmer) diluted in DELFIA assay buffer (PerkinElmer) was added for 30 min and shaken at slow speed at room temperature. After washing for 6 times, DELFIA Enhancement Solution was added for 1 h until the optimal signal was reached. Time-resolved fluorescence was measured using the BMG reader (BMG Labtech, Cary, NC). The experiment was performed in triplicates. As negative control only biotinylated APO peptide was incubated with small molecules, without APS3v-HLA-DR3. Percent inhibition was calculated by the following formula: 100-100 X [APS3v-HLA-DR3-APO-small molecule/APS3v-HLA-DR3-APO (no small molecule)].

### 2.5 Cepharanthine (S53) and Other Small Molecules

Cepharanthine (S53) used in our studies was purchased as a beige powder from Microsource Discovery Systems (Gaylordsville, CT). Identity and purity of the sample was verified (15). Other small molecules used in this study were purchased from ChemBridge (San Diego, CA).

### 2.6 Dose Response of Cepharanthine Blocking Peptide Binding to APS3v-HLA-DR3

0.012 mg/ml of recombinant APS3v-HLA-DR3 was incubated with 10 µM biotinylated peptides [APO, Tg.1571, GAD.492, or TPO.758 (Genscript)], together with decreasing doses of Cepharanthine (0.4 mM, 0.2 mM, 0.1 mM, 0.05 mM), for 48 h at 37°C in a binding buffer. *In vitro* binding assay was performed as described above for accessing the percent inhibition.

### 2.7 Mice

Female humanized NOD-DR3 mice used in this study are knockout for murine MHC class II and express DRB1*0301 (17) and confirmed by sequencing to contain the 4 critical amino acids of the APS3v-HLA-DR3 pocket (10). The derivation and genotyping of the mice were described as previously (15).

### 2.8 Induction of T-Cell Response to Islet and Thyroid Autoantigens in Humanized NOD-DR3 Mice as a Model of APS3v

Previously we have identified 4 thyroid and islet peptides that triggered T-cell responses in NOD-DR3 mice – Tg.1571, GAD.492, TPO.338 and TPO.758 (12). However, only 3 of these peptides (Tg.1571, GAD.492, and TPO.758) elicited significant B-cell (antibody) responses (**Supplementary Figure 1**). Therefore, we concluded that these 3 peptides are the major T-cell epitopes in APS3v and used them to induce a model of APS3v in humanized NOD-DR3 mice. To induce the APS3v model, 22 female NOD-DR3 mice 4-6 weeks old were co-immunized subcutaneously with these three peptides: Tg.1571, GAD.492 and TPO.758 (100 µg each peptide), in Complete Freund’s Adjuvant (CFA, Sigma-Aldrich) as previously described (12, 15). Mice were immunized with peptides on day 0 and boosted on day 7, then sacrificed on day 21. As negative control, humanized NOD-DR3 littermates were immunized with PBS emulsified in CFA.

### 2.9 Lymphocytes Isolation

Spleen and draining lymph nodes were collected from mice upon sacrifice. Lymphocytes were isolated as previously described (12, 15). Briefly, the spleens and draining lymph nodes were harvested in complete RPMI (Corning, NY) supplemented with 10% FBS (Sigma-Aldrich) and 1 mM sodium pyruvate (Sigma-Aldrich). They were cut and pressed in circular motion using a plunger from a 10 ml syringe. The suspension was filtered through a 100 µm cell strainer twice and centrifuged 200 x g for 10 min. The pellet was washed with RPMI and centrifuged one more time. 5 ml Ammonium-Chloride-Potassium (ACK) lysis buffer was added to remove erythrocytes from the spleen. After 5-min incubation with ACK lysis buffer at room temperature with occasional shaking, cells were centrifuged at 200 x g for 10 min. The pellet was resuspended in RPMI and the cells were counted and plated.

### 2.10 Cytokine Assays

Milliplex mouse cytokines/chemokine magnetic panel (Catalog no. MCYTOMAG-70K, EMD Millipore Corporation, Billerica, MA) was used to assay the cytokines, as previously described (12, 15, 18). Briefly, splenocytes were plated at 2 x 10^6^ cells per well in 500 µl of medium (RPMI/10% FBS). Supernatants from stimulated lymphocytes were collected 48 h after stimulation with peptides and stored in -80°C until the assay was performed. The 96-well plate supplied in the kit was washed with the wash buffer supplied and the plate was shaken for 10 min at room temperature. Standards and quality controls were added, followed by the samples. The pre-mixed beads (Interferon gamma and IL-2) were sonicated, vortexed and added to the wells. After shaking the plate overnight at 4°C, the plate was washed twice with wash buffer. Detection antibodies were added for 1 h at room temperature, and Streptavidin-Phycoerythrin was added for 30 min at room temperature. The plate was washed twice and sheath fluid was added to resuspend the beads for 5 min before reading in Luminex 200 with xPONENT software (Luminex, Austin, Texas).

### 2.11 Purification of Mouse Thyroglobulin (Tg)

Mouse thyroglobulin was purified using a modified procedure (13). Briefly, mouse thyroid lysate in 10 mM HEPES pH 7.4, 100 mM NaCl buffer was loaded onto a 5 mL HiTrap Q anion exchange column and was run at 4 ml/min, using a linear gradient from 0.1 to 1 M NaCl. Mouse Tg eluted in a peak spanning from 270 to 440 mM NaCl.

### 2.12 Autoantibody Measurements

Sera were collected from mice upon sacrifice and stored in -20°C. Nunc Maxisorp ELISA plate (Thermo Fisher Scientific, Waltham, MA) was coated with 10 µg/ml of either Tg.1571, GAD.492, TPO.758 or mouse Tg in bicarbonate buffer (Sigma-Aldrich) at pH 9.6 and incubated overnight. The ELISA plate was washed 4 times with PBS supplemented with 0.05% Tween (Sigma-Aldrich) [PBST]. After blocking with 2.5% BSA in PBST for 1 h at 37°C, the plate was washed with PBST for 6 times. 100 µl of the diluted sera (1:100 in 0.5% BSA/PBS) was added for 2 h with slow shaking at room temperature. After washing for 6 times with PBST, anti-mouse IgG secondary antibody (Sigma-Aldrich) was added at 1:30,000 dilution in 1% BSA/PBST and incubated for 30 min at 37 °C. After washing the plate for 4 times with PBST, the freshly prepared *para*-nitrophenylphosphate substrate (Sigma-Aldrich) was added for 1 h and the plate was read at 405 nm using the BMG reader (BMG Labtech, Cary, NC).

GAD antibody ELISA was performed using Glutamic Acid Decarboxylase (GAD) Autoantibody ELISA kit (Kronus, Star, ID). Briefly, standards, controls and serum samples were added to wells pre-coated with GAD and incubated for 1 h with shaking. After three washes using wash buffer supplied in the kit, GAD65-biotin was added for 1 h with shaking. Followed by another three washes, streptavidin-peroxidase was added for 20 min with shaking. After 3 more washes with wash buffer and one wash with deionized water, peroxidase substrate was added for 20 min, followed by stop solution. Absorbance at 450 nm was measured using the BMG reader (BMG Labtech, Cary, NC).

### 2.13 Free T4 Measurements

Sera were collected from mice and were assayed for free thyroxin (fT4) measurements using free thyroxine ELISA kit (Alpha Diagnostic International, San Antonio, TX, catalog no. 1110). Assay was performed according to manufacturer’s protocol. The standards were run simultaneously with the serum samples to obtain a standard curve. Briefly, serum samples were added to the wells pre-coated with T4-specific antibodies. Diluted enzyme conjugate was added for 1 h at 37 °C, followed by three washes using the wash buffer supplied in the kit. TMB substrate was added for 15 min at 37°C, followed by stop solution. Absorbance at 450 nm was measured using a BMG ELISA reader (BMG Labtech).

### 2.14 Small Molecule Inhibition of Cytokine Production *Ex Vivo*

Eight NOD-DR3 mice were immunized with the three thyroidal/islet peptides. Upon sacrifice, splenocytes were incubated with 1 µM of small molecules (S5, S9, S15, S53) together with TPO.758 (20 µg/ml), GAD.492 (20 µg/ml) or Tg.1571 (20 µg/ml) respectively to assess the blockade of these peptides’ presentation by the tested small molecules. Supernatants from stimulated lymphocytes were collected 48 h after stimulation with peptides and small molecules, and stored in -80 °C until the cytokine assay was performed.

### 2.15 Blocking the Induction of APS3v by Cepharanthine *In Vivo*

Four humanized NOD-DR3 mice were injected with 125 µg of Cepharanthine intraperitoneally (IP) on days -2 and -1 prior to the immunization with the peptide mix of Tg.1571, GAD.492 and TPO.758 on day 0; mice were injected with Cepharanthine IP again on days 5 and 6 prior to immunization with the same peptide mix on day 7. As controls, four humanized NOD-DR3 littermates were injected with the vehicle that was used to dissolve Cepharanthine for *in vivo* studies [ethanol: PEG400 (Fisher Scientific): saline (5:20:75)] (Frontage Laboratories, Exton, PA) using the same timeline for immunizations.

### 2.16 T-Cell Stimulation and CFSE Analysis

Cells harvested from the spleen and draining lymph nodes of immunized mice were resuspended at 2 X 10^6^ cells/ml in 0.1% BSA/PBS. 1 X 10^6^ cells were labeled with 1.5 µM carboxyfluorescein diacetate succinimidyl ester (CFSE) (Life Technologies). After incubating for 10 min at 37 °C, the staining was terminated by the addition of 4 volumes of ice-cold RPMI with 10% FBS. After incubating on ice for 5 min, the cells were washed three times with fresh RPMI and resuspended in fresh medium for counting.

The CFSE-labeled cells were plated at 2 X 10^5^ cells/well in 100 µl medium (RPMI, 10% FBS). The cells were incubated with: medium, Tg.1571, GAD.492, TPO.758 (20 µg/ml), a negative control peptide (NC) (20 µg/ml), or mouse anti-CD3/CD28 beads (Life Technologies) that activate all CD4+ T-cells non-specifically, as a positive control. The cells were collected after 5 days for flow cytometry analysis. All experiments were performed in quadruplicates. The results were analyzed using Flowjo (Tree Star, Ashland, OR). The stimulation index was calculated by using the following formula: stimulation index = [% proliferating lymphocytes (peptide/mitogen-treated)]/[% proliferating lymphocytes (medium-treated)].

### 2.17 Histology

Thyroid glands and pancreases were dissected and removed from immunized NOD-DR3 mice after sacrifice and stored in 10% formaldehyde until processing. After creating paraffin tissue blocks, thyroids and pancreases were sectioned and stained with hematoxylin-eosin for histological analysis (Histology and Comparative Pathology Core, Albert Einstein College of Medicine).

### 2.18 MD Simulations of the Complex of Cepharanthine With APS3v-HLA-DR3

**Figure 2:**
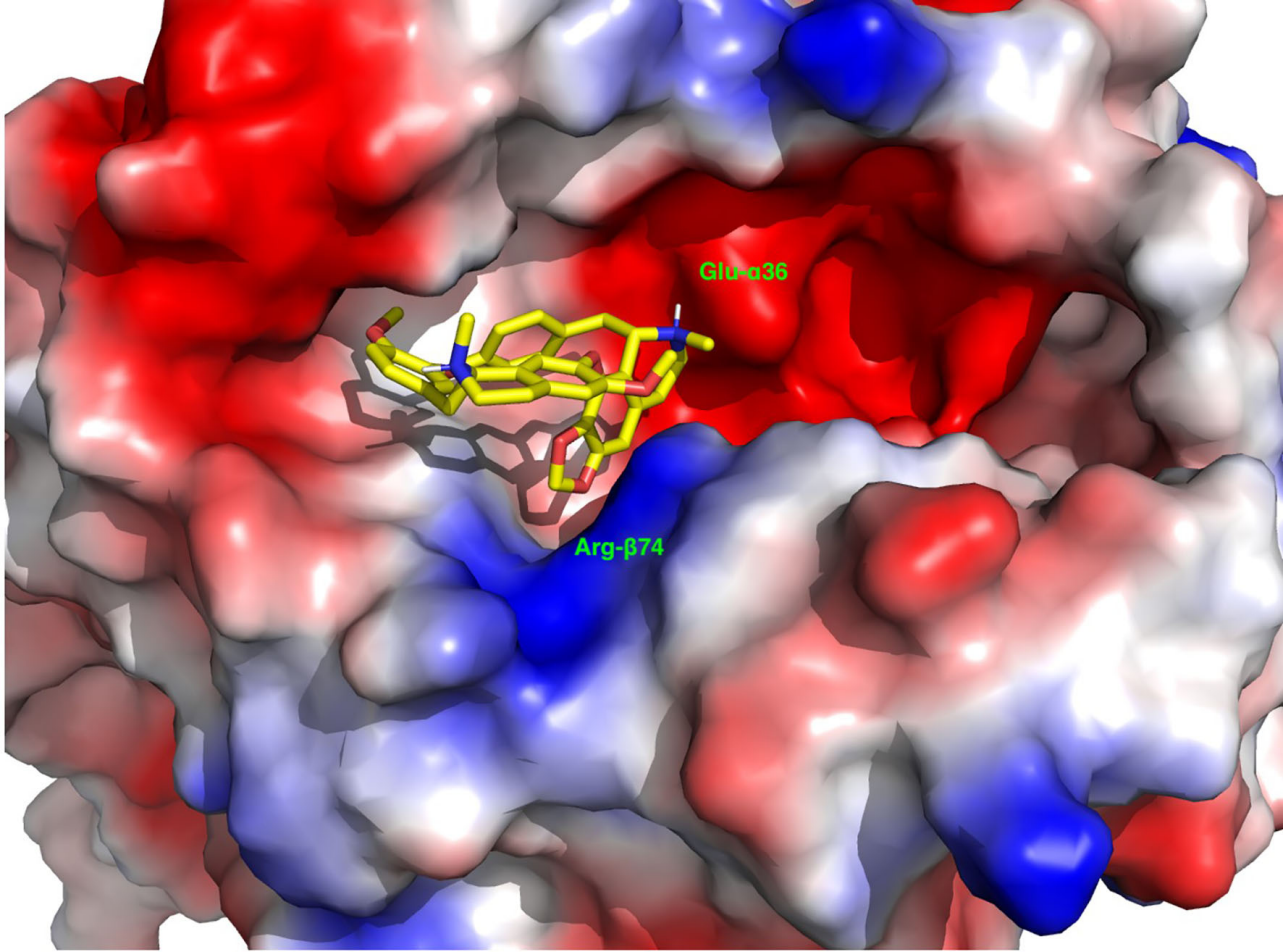
A representative depiction of the binding of Cepharanthine to the binding pocket of the APS3v-associated HLA-DR3. The surface is colored by the electrostatic potential, where blue indicates a positive and red a negative electrostatic potential. Note the anchoring of the methylenedioxy group next to the Arg-β74 (blue) and the positively charged methyl-piperidine group next to Glu-α36.

The docked structure of the Cepharanthine in the APS3v-HLA-DR3 was used to construct the system for MD simulations. The system was placed in a truncated octahedral box filled with waters and ions with walls at a distance of 10 Å from the solute. The solute was positionally restrained and the system was minimized, heated and equilibrated by gradually relaxing the restraints. A production run at NPT was conducted for 1 μs. The trajectory was written to produce 50,000 structures. The stripped trajectory was clustered by 2D-rms to produce three clusters: the first was a transient structure for the first 6.5 ns and the other two represent two clusters in which Cepharantine undergoes a minor conformational change. These two clusters were distributed at a ratio of 3:1. We selected the cluster center of the larger cluster as a representative structure of the complex between Cephranthine and the APS3v-HLA-DR3 (**Figure 2**).

### 2.19 Statistical Analysis

Prism 5 software was used to perform the statistical analysis. Student’s t test (unpaired t test, one-tailed) was used for comparisons of means of the experimental vs. control groups for each of the continuous variables measured. A *p* value < 0.05 was considered statistically significant.

### 2.20 Study Approval

The project was approved by the Institutional Animal Care and Use Committees (IACUC) of the Icahn School of Medicine at Mount Sinai and Albert Einstein College of Medicine. All animal studies performed at Mount Sinai and Einstein were carried out according to the guidelines of the IACUC’s of the Icahn School of Medicine at Mount Sinai and Albert Einstein College of Medicine, respectively.

## 3 Results

### 3.1 Virtual Screening of Small Molecules Blocking APS3v-HLA-DR3

Our virtual screen identified 100 small molecules that were predicted to block the unique APS3v-HLA-DR3 pocket. To produce a manageable selection of compounds for validation we clustered the 100 compounds by their simplified molecular input line entry system (SMILES) similarity into 20 clusters. Of the 20 clusters we selected the top scoring members in each cluster. We have enhanced this list to include a total of 11 compounds that blocked APS3v-HLA-DR3, the key HLA-DR3 pocket that triggers APS3v.

### 3.2 *In Vitro* Testing of 11 Small Molecules Identified by the Virtual Screen

**Figure 3:**
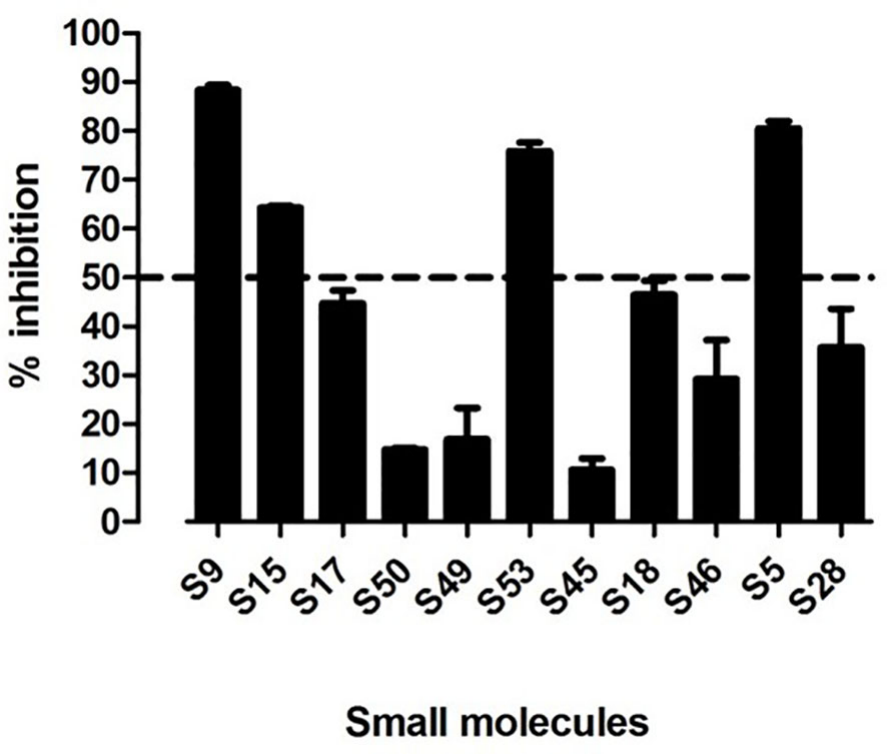
11 top scoring small molecules were tested for blocking APO peptide binding to APS3v-HLA-DR3 in ELISA. **Figure 3** shows the *in vitro* screening results of the 11 top scoring small molecules identified in the virtual screen. 4 small molecules (S9, S15, S53 and S5) inhibited APO peptide binding to APS3v-HLA-DR3 by more than 50%.

We tested the selected 11 small molecules identified by the virtual screen in our *in vitro* ELISA, to test whether the 11 small molecules can block APO peptide (the strongest known peptide binder to HLA-DR3) binding to recombinant APS3v-HLA-DR3. Briefly, recombinant APS3v-HLA-DR3 was incubated with biotinylated APO peptide together with the 11 small molecules, for 48 h at 37 °C in a binding buffer. A 96-well ELISA plate was coated overnight with L243 antibody that specifically recognizes the DRα chain of HLA-DR when it is properly folded and complexed with the β chain. After washing and blocking, 100 µl of the pre-incubated mix (containing recombinant APS3v-HLA-DR3 protein, APO, and small molecules) were added onto the plate and shaken at slow speed at room temperature. DELFIA Europium-labeled streptavidin diluted in DELFIA assay buffer was added and shaken at slow speed at room temperature. After washing, DELFIA Enhancement Solution was added until the optimal signal was reached. Time-resolved fluorescence was measured using the BMG reader. Percent inhibition was calculated by the following formula: 100-100 X [APS3v-HLA-DR3-APO-small molecule/APS3v-HLA-DR3-APO (no small molecule)] (see *Materials and Methods* for details). Of these, 4 small molecules (S5, S9, S15 and S53 that belonged to the original 11 compounds identified as binding to HLA-DRβ-Arg74) showed >50% inhibition (**Figure 3**). S53 is our code designation for Cepharanthine, which we have previously shown to be effective in blocking experimental autoimmune thyroiditis (EAT) and experimental autoimmune Graves’ disease (EAGD) (15, 18).

### 3.3 Validating That the 4 Small Molecule Hits Block Thyroid/Islet Peptides *In Vitro*

**Figure 4:**
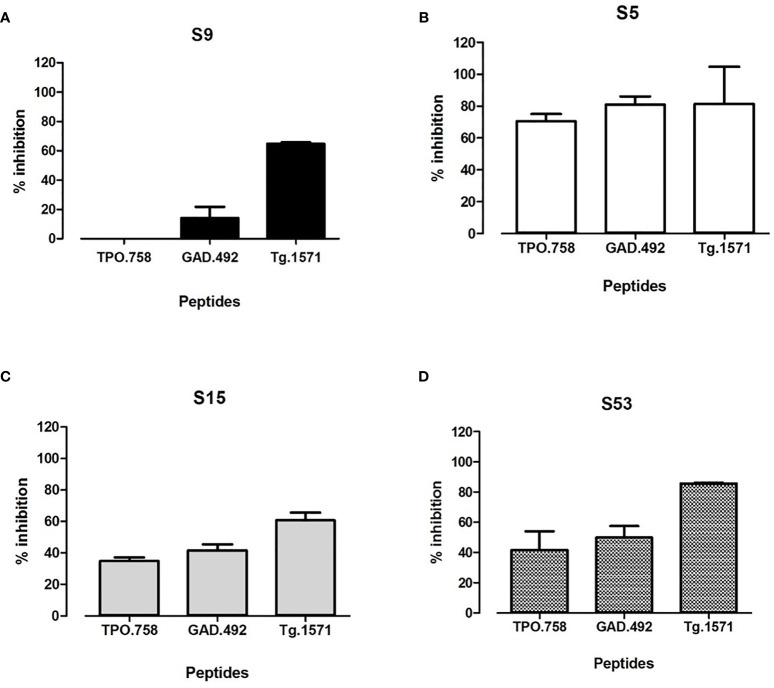
The top 4 hit small molecules (S9, S5, S15, S53) were tested for blocking biotinylated TPO.758, GAD.492 and Tg.1571 binding to APS3v-HLA-DR3 in ELISA. **Figure 4** shows the ELISA results of blocking TPO.758, GAD.492 and Tg.1571 binding APS3v-HLA-DR3 by S9 **(A)**, S5 **(B)**, S15 **(C)** and S53 **(D)**.

To test if the 4 small molecule hits showing inhibition of APO binding to APS3v-HLA-DR3 can block thyroid and islet peptides binding to APS3v-HLA-DR3, we incubated recombinant APS3v-HLA-DR3 protein with biotinylated TPO.758, GAD.492, or Tg.1571, either alone or together with each of the 4 small molecules individually. ELISA was performed as described above for APO peptide. S5, S15 and S53 blocked the binding of all three peptides to APS3v-HLA-DR3, but S9 blocked only Tg.1571 and GAD.492 (**Figure 4**).

### 3.4 S53 Blocked Peptide Binding to APS3v-HLA-DR3 *In Vitro* in a Dose-Dependent Manner

**Figure 5:**
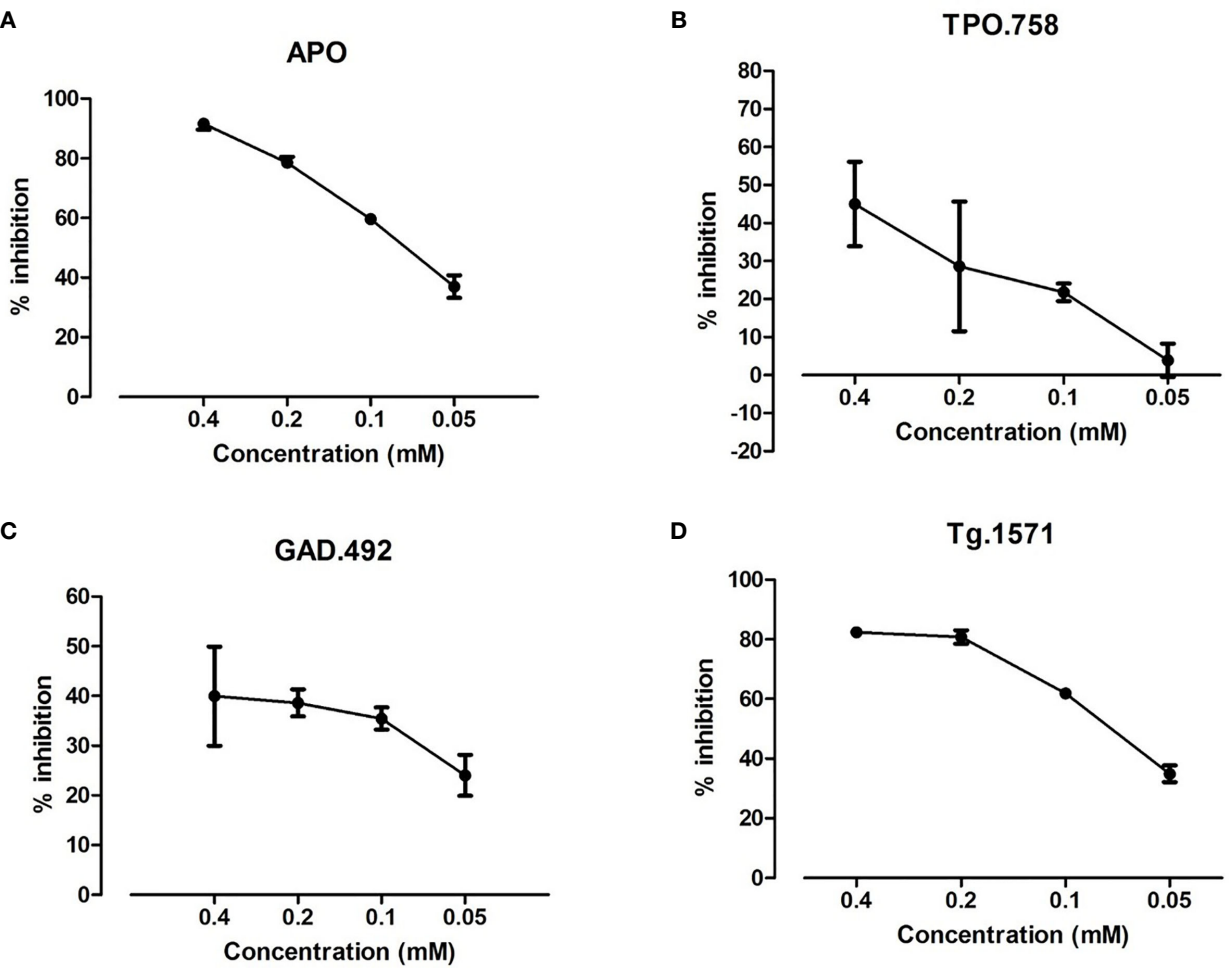
S53 at decreasing doses (0.4 mM, 0.2 mM, 0.1 mM, 0.05 mM) was tested for blocking the binding of biotinylated APO, TPO.758, GAD.492 and Tg.1571 to APS3v-HLA-DR3 by ELISA. The ELISA results showed that of S53 blocked APO **(A)**, TPO.758 **(B)**, GAD.492 **(C)** and Tg.1571 **(D)** in a dose-dependent manner.

Of the 4 validated small molecules we selected S53 for further testing because it is the only one that has been used in humans before. Recombinant APS3v-HLA-DR3 was incubated with biotinylated APO, TPO.758, GAD.492 or Tg.1571, either alone or with decreasing doses (0.4 mM, 0.2 mM, 0.1 mM, 0.05 mM) of S53 [which we have studied previously (15, 18)]. ELISA was performed as previously described. S53 blocked the binding of each peptide in a dose-dependent manner (**Figure 5**).

### 3.5 Generating a Humanized Mouse Model Of APS3v Showing Autoimmune Responses to Thyroid and Islet Major Autoantigens

#### 3.5.1 T-Cell Responses in Humanized NOD-DR3 Mice Co-Immunized With Tg.1571, GAD.492 and TPO.758 Peptide Mix

**Figure 6:**
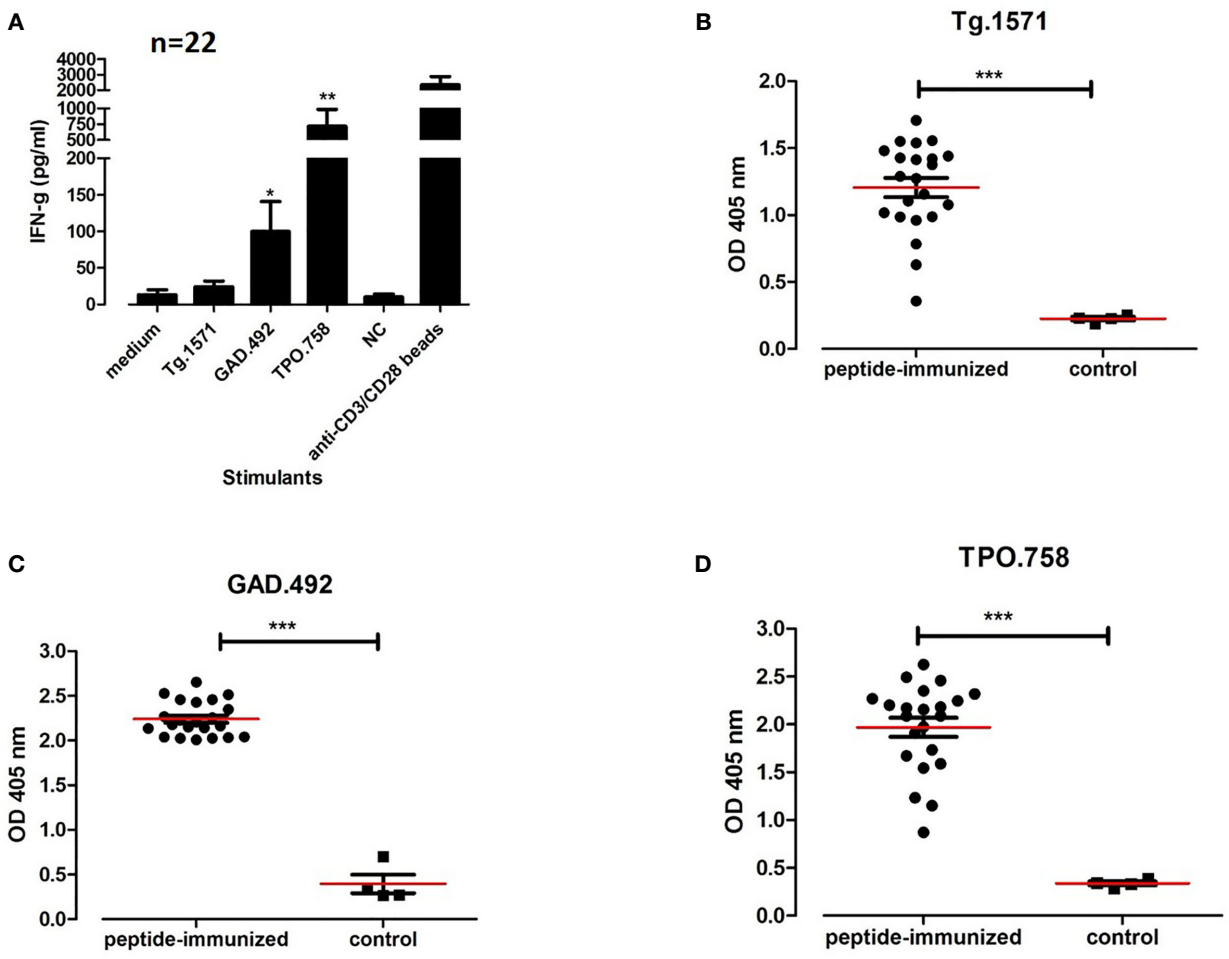
Twenty-two humanized NOD-DR3 mice were co-immunized with Tg.1571, GAD.492 and TPO.758. Supernatant from stimulated splenocytes were collected and assayed for cytokine production. Sera were also collected for autoantibody measurements. **Figure 6** shows interferon gamma production **(A)** and autoantibody response to Tg.1571 **(B)**, GAD.492 **(C)** and TPO.758 **(D)** of humanized NOD-DR3 mice co-immunized with the three thyroid and islet peptides. NC, negative control peptide. Anti-CD3/CD28 beads, positive control. Control mice were immunized with PBS and adjuvant. *p < 0.05; **p < 0.01; ***p < 0.001.

Twenty-two humanized NOD-DR3 mice were co-immunized with a peptide mix of Tg.1571, GAD.492 and TPO.758 together with adjuvant. Splenocytes and draining lymph node cells of immunized mice were isolated and stimulated with either: (1) Tg.1571, GAD.492, or TPO.758, thyroid and islet peptides that were previously shown to bind strongly to APS3v-HLA-DR3 (12); (2) unrelated peptide (negative control); or (3) mouse anti-CD3/CD28 beads (positive control), for 48 hours to test for T-cell activation of cytokine responses using the Milliplex mouse cytokines/chemokine magnetic panel from EMD Millipore (see *Materials and Methods* for details). Lymphocytes isolated from mice co-immunized with Tg.1571, GAD.492 and TPO.758 showed strong interferon gamma (IFN-g) responses to GAD.492 (99.6 pg/ml) (*p*<0.05), TPO.758 (716.62 pg/ml) (*p*<0.01), and Tg.1571 (24.2 pg/ml) (*p*=0.1511), compared to the control (**Figure 6A**).

#### 3.5.2 Humanized NOD-DR3 Mice Co-Immunized With Tg.1571, GAD.492 and TPO.758 Developed Antibodies to Thyroid/Islet Peptides and Mouse Tg

**Figure 7:**
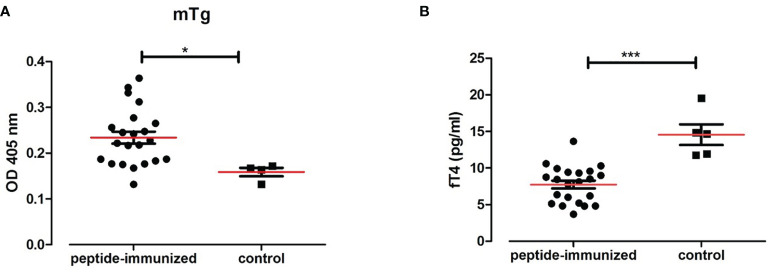
Twenty-two humanized NOD-DR3 mice were co-immunized with Tg.1571, GAD.492 and TPO.758. **Figure 7** shows the humoral response and thyroid functions in humanized NOD-DR3 mice co-immunized with Tg.1571, GAD.492 and TPO.758. Autoantibody response to mouse Tg **(A)** and free T4 levels **(B)** of humanized NOD-DR3 mice co-immunized with the three thyroid and islet peptides, compared to control mice immunized with PBS and adjuvant. *p < 0.05; ***p < 0.001.

Sera from humanized NOD-DR3 mice co-immunized with Tg.1571, GAD.492 and TPO.758 (n=22) were collected and stored in -20°C until ELISA was performed for antibody measurements. Sera was added to ELISA plates coated with each peptide (Tg.1571, GAD.492 and TPO.758) respectively and incubated for 2 h before anti-mouse IgG secondary antibody and *para*-nitrophenylphosphate substrate were added for measurements using the BMG reader (see *Materials and Methods* for details). Humanized NOD-DR3 mice co-immunized with Tg.1571, GAD.492 and TPO.758 developed a strong humoral antibody response to each of the peptides (*p*<0.001, compared to PBS-immunized controls) (**Figures 6B–D**). The immunized NOD-DR3 mice also developed high levels of antibodies against mouse Tg protein (*p*<0.05, compared to controls) (**Figure 7A**). We also checked if the mice developed antibodies against GAD protein. Two out of 22 immunized mice developed antibodies against GAD protein while none of the controls developed GAD antibodies (data not shown).

#### 3.5.3 Free T4 Levels in Humanized NOD-DR3 Mice Co-Immunized With Tg.1571, GAD.492 and TPO.758

Twenty-two NOD-DR3 mice co-immunized with Tg.1571, GAD.492 and TPO.758 with adjuvant showed significantly lower free T4 levels (7.77 pg/ml) compared to control NOD-DR3 mice immunized with PBS and adjuvant (14.84 pg/ml) (*p*<0.001) (**Figure 7B**).

### 3.6 Thyroid and Pancreatic Histology

Upon co-immunization of thyroid (Tg.1571, TPO.758) and islet (GAD.492) peptides, thyroid or islet lymphocytic infiltration was not observed in the 22 humanized NOD-DR3 mice (data not shown).

### 3.7 Cepharanthine (S53) Blocks T-Cell Activation by Thyroid and Islet Peptides *Ex Vivo*

**Figure 8:**
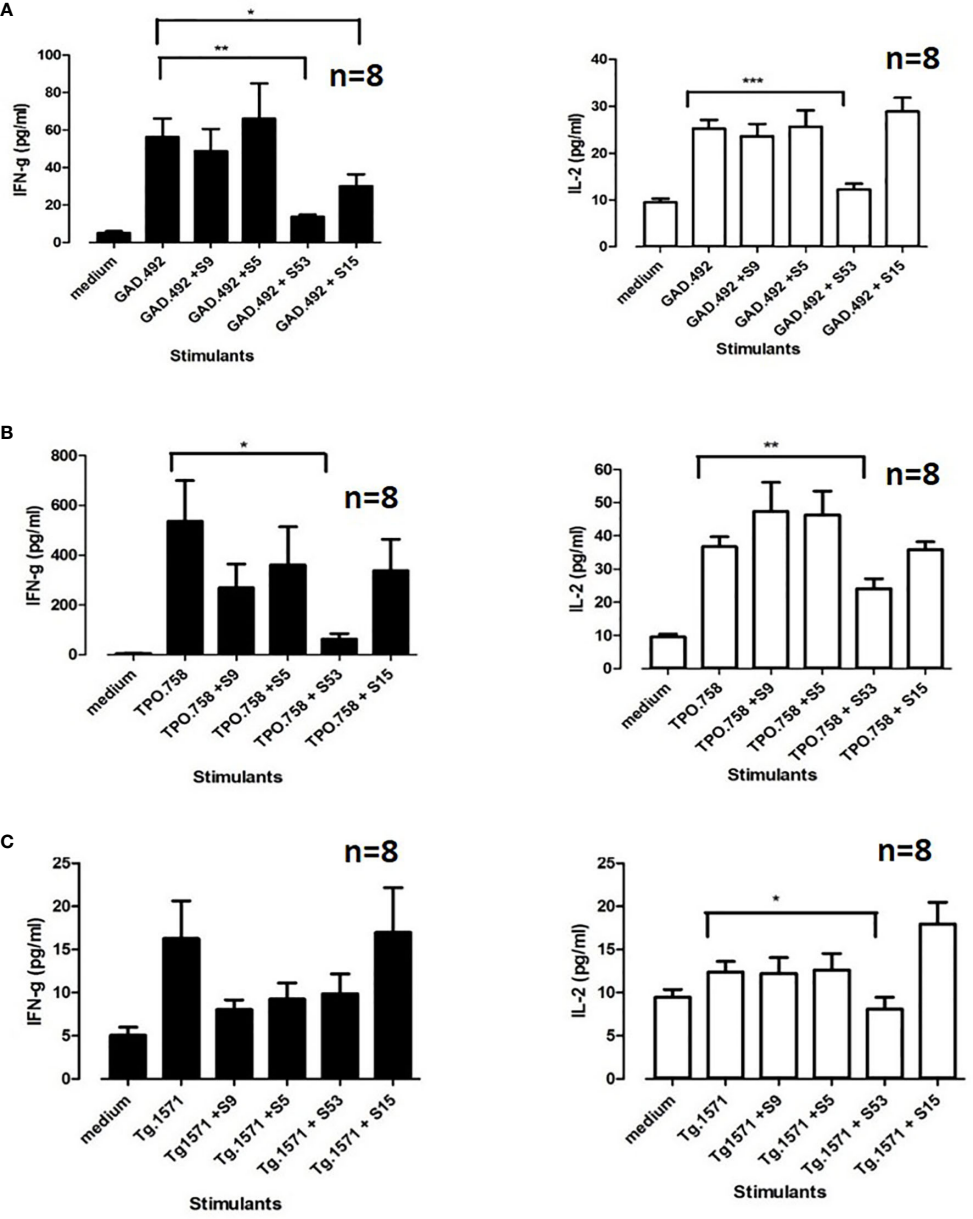
Eight humanized NOD-DR3 mice were co-immunized with Tg.1571, GAD.492 and TPO.758. Splenocytes were co-incubated with peptides and small molecules *ex vivo*. Stimulated splenocytes were collected and assayed for cytokine production. **Figure 8** shows interferon gamma and IL-2 production when GAD.492 **(A)**, TPO.758 **(B)** and Tg.1571 **(C)** were co-incubated with the top four small molecules (S9, S5, S53 and S15) in splenocytes of the immunized mice. *p < 0.05; **p < 0.01; ***p < 0.001.

Upon sacrifice of the 8 NOD-DR3 mice co-immunized with Tg.1571, GAD.492 and TPO.758, their splenocytes and draining lymph node cells were harvested and stimulated with Tg.1571, GAD.492 or TPO.758, in the presence or absence of each of the 4 small molecule hits (S5, S9, S15 and S53) to assay for cytokine production using the Milliplex mouse cytokines/chemokine magnetic panel from EMD Millipore (see *Materials and Methods* for details). Both S15 and S53 significantly reduced GAD.492-induced IFN-g production [reduced from 56.3 pg/ml (GAD.492) to 30.1 pg/ml (GAD.492 + S15, *p*<0.05) and 13.5 pg/ml (GAD.492 + S53, *p*<0.01)]. Interestingly, only S53 reduced GAD.492-induced IL-2 production (reduced from 25.2 pg/ml to 12.3 pg/ml, *p*<0.001) (**Figure 8A**). Only S53 significantly inhibited TPO.758-induced IFN-g (from 535.6 pg/ml to 62.3 pg/ml, *p*<0.05) and IL-2 (from 36.7 pg/ml to 24.1 pg/ml, *p*<0.01) production (**Figure 8B**). Similarly, only S53 significantly inhibited Tg.1571-induced IL-2 production, from 12.4 pg/ml to 8.1 pg/ml (*p*<0.05) (**Figure 8C**), albeit it did not significantly reduce IFN-g production in response to Tg.1571.

### 3.8 Cepharanthine (S53) Blocks Activation of T-Cells to Thyroid and Islet Peptides *In Vivo*

**Figure 9:**
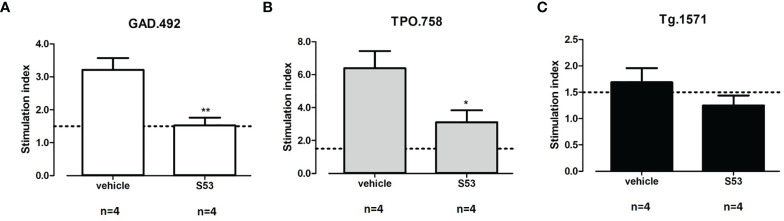
Four humanized NOD-DR3 mice were co-immunized with Tg.1571, GAD.492 and TPO.758 and treated with Cepharanthine (S53) or vehicle. Stimulated splenocytes were collected and T-cell proliferation assay was performed. **Figure 9** shows the stimulation index in response to GAD.492 **(A)**, TPO.758 **(B)** and Tg.1571 **(C)**. *p < 0.05; **p < 0.01.

Four humanized NOD-DR3 mice were treated with Cepharanthine (S53) or vehicle (used to dissolve Cepharanthine) for 2 consecutive days before each of the two immunizations with Tg.1571, GAD.492 and TPO.758 peptide mix. At sacrifice, splenocytes and draining lymph node cells were incubated with each peptide and labeled with CFSE for 5 days for T-cell stimulation analysis. Briefly, the CFSE-labeled cells were plated at 2 X 10^5^ cells/well in 100 µl medium (RPMI, 10% FBS). The cells were incubated with: medium, Tg.1571, GAD.492, TPO.758 (20 µg/ml), a negative control peptide (NC) (20 µg/ml), or mouse anti-CD3/CD28 beads (Life Technologies) that activate all CD4+ T-cells non-specifically, as a positive control. The cells were collected after 5 days for flow cytometry analysis (see *Materials and Methods* for details). S53 significantly suppressed T-cell proliferation induced by GAD.492 and TPO.758 compared to vehicle control; the stimulation index decreased from 3.21 to 1.53 for GAD.492 (*p*<0.01) (**Figure 9A**) and from 6.39 to 3.11 for TPO.758 (*p*<0.05) (**Figure 9B**). S53 also suppressed T-cell proliferation induced by Tg.1571, although not statistically significant (**Figure 9C**). S53 did not significantly affect the free T4 levels or antibody levels against mouse Tg (data not shown).

## 4 Discussion

APS3v is a variant of APS3 which consists of AITD and T1D developing in the same individual (5). To identify new therapies for APS3v we developed a novel humanized mouse model of APS3v. In this model we immunized humanized NOD-DR3 mice, which carry the human APS3v-HLA-DR3 pocket, with the 3 key thyroid and islet peptides that trigger APS3v (12). Following immunization, the mice developed cellular and humoral autoimmune responses to thyroidal and islet antigens, as well as biochemical hypothyroidism, albeit they did not develop histological signs of thyroiditis and insulitis.

We performed a virtual screen of a large library of compounds followed by *in vitro* confirmation of hit compounds, as well as *ex vivo* and *in vivo* testing in our new APS3v mouse model, to identify small molecules that can suppress APS3v. Cepharanthine (S53) showed activity in all assays suggesting that it may be effective in APS3v patients. Cepharanthine (S53) is a plant alkaloid extracted from *Stephania cepharantha Hayata*. It has been used in Japan for more than 40 years for a wide variety of chronic and acute diseases (19–21). Cepharanthine can be given by oral or parenteral administration (19–21). Some of the clinical conditions in which Cepharanthine has been used include alopecia areata (22, 23), chemotherapy induce leukopenia (20) and other medical conditions (24–29). Cepharanthine was also reported to have other effects including anti-tumor activity (30–32), reversal of multiple drug resistance (33, 34) and anti-allergic actions (35, 36). No major side effects were reported, except for rare and reversible elevation of liver enzymes (37), although it can be due to the limited number of studies.

Previously our lab has established two new AITD mouse models for Hashimoto’s thyroiditis (HT) and Graves’ disease (GD) using humanized DR3 mice (15, 18). In both models we reported that Cepharanthine blocked the disease *ex vivo* and *in vivo* (15, 18). In the current study we discovered that Cepharanthine may also be effective in APS3v in addition to AITD.

What is the reason that Cepharanthine can block different antigens from binding to HLA-DR3 in both AITD (HT and GD) and APS3v mouse models? We hypothesize that Cepharanthine is very effective in blocking specifically the HLA-DR3 pocket that is associated with both AITD and APS3v. Indeed, we have previously shown that Cepharanthine did not block the HLA-DR2 pocket (15). Therefore, it is possible peptides, thyroidal or islet derived, that are presented within the HLA-DR3 pocket, but not peptides that are presented within other MHC class II pockets (DQ and DP pockets, as well as non-DR3 HLA-DR pockets), can be blocked by Cepharanthine. We previously examined the binding of Cepharanthine to the HLA-DR3 pocket and revealed that Cepharanthine interacts directly with DRβ1-Arg74, which is the key pocket amino acid both in AITD and in APS3v (10, 15, 38). Furthermore, the amino acids substituted in APS3v do not interact directly with Cepharanthine, thus the ability of Cepharanthine to block both the AITD and APS3v-HLA-DR3 pockets is consistent with our computational studies showing its interaction with the residues shared by both pockets and not with those that are unique to each pocket. For example, the shortest distance is between Tyr-β47 and it is on the average 14Å. The binding of Cepharanthine to the APS3v-HLA-DR3 pocket is shown in **Figure 2**. Similar to its binding to the AITD HLA-DR3 pocket here also Cepharanthine is anchored between Arg-β74 (highlighted by the positive potential surface, blue) and Glu-α36 (highlighted by the negative potential surface, red). At the other end there are hydrophobic interaction with phenylalanines and aliphatic amino acids. Overall, the binding of Cepharanthine to the APS3v-associated DR3 pocket is very similar to its binding to the AITD-associated DR3 pocket. This provides a reasonable explanation for its effectiveness in both our AITD and APS3v mouse models.

Other than APS3v, there are other autoimmune diseases that are associated with HLA-DR3, including Graves’ disease, Hashimoto’s thyroiditis, Addison’s disease, type 1 diabetes and systemic lupus erythematosus (39). Therefore, Cepharanthine may have clinical efficacy in these autoimmune diseases too if the patient is positive for HLA-DR3. HLA-DR3 is also a susceptibility gene for APS2 (which includes T1D, AITD and Addison’s disease), even though APS2 has a different HLA class II associations than APS3v (40). We have shown that Cepharanthine is highly effective *in vitro* and in mouse models against three autoimmune endocrine conditions that are triggered by HLA-DR3, namely HT (15), GD (18), and APS3v. Therefore, it is possible that Cepharanthine may be effective against other autoimmune conditions triggered by HLA-DR3. Specifically, recent data suggest that T1D may have two subtypes, one that is associated with HLA-DR4, younger age of onset, and the development of insulin antibodies, and another that is associated with HLA-DR3, older age of onset, and with the development of GAD antibodies (41–45). Therefore, it is possible that Cepharanthine may be effective against the subtype of T1D that is associated with HLA-DR3 and the production of GAD antibodies. Indeed, our data showed that Cepharanthine blocked the GAD.492 peptide from binding to HLA-DR3. One limitation of our study is that we used humanized mice carrying only the human HLA-DR3, but with no DQ or DP. Since most patients with APS3v carry HLA-DQ2 or DQ8, which confer significant disease risk for T1D, it remains to be determined whether blocking of DQ in addition to blocking HLA-DR3 will also be needed to prevent and/or treat APS3v.

Blocking HLA class II pockets is emerging as a new and promising therapeutic approach in autoimmunity. Recently, it was reported that methyldopa was able to specifically block HLA-DQ8 in patients with recent onset T1D and suppress T-cell responses to insulin (46). In addition, our group has reported that two retro-inverso-D (RID) - amino acid peptides, RI-EXT and RI-CT, were able to inhibit insulin peptide binding to HLA-DQ8 and its presentation to T-cells in T1D (47). This study and our previous studies suggest that Cepharanthine may be a promising compound that can block HLA-DR3 in AITD and T1D.

In summary, we developed a novel mouse model of APS3v by co-immunizing humanized NOD-DR3 mice with thyroid and islet peptides. Using this mouse model we showed that Cepharanthine can block specific thyroid and islet peptides binding to the APS3v-HLA-DR3 pocket and inhibit T-cell activation in the model. These new data, as well as our previous findings that Cepharanthine is effective in animal models of HT and GD suggest that Cepharanthine may be an effective compound in several autoimmune endocrine disorders that are associated with HLA-DR3. Further testing of Cepharanthine in APS3v patients carrying the specific APS3v-HLA-DR3 pocket will provide insight on the utility of Cepharanthine in human disease.

## Data Availability Statement

The original contributions presented in the study are included in the article/**Supplementary Material**. Further inquiries can be directed to the corresponding author.

## Ethics Statement

The animal study was reviewed and approved by Institutional Animal Care and Use Committees (IACUC) of the Icahn School of Medicine at Mount Sinai and Albert Einstein College of Medicine.

## Author Contributions

CL and FM conducted most of the experiments and analyzed the results. YT conceived the idea for the project and participated in data analysis and interpretation. RO performed the virtual screen and helped drafting the manuscript. HH provided assistance for managing the mouse colony. LF, KK, and OC prepared the mouse thyroglobulin. CL and YT wrote the manuscript. All authors contributed to the article and approved the submitted version.

## Funding

This work was supported in part by grants DK067555 and DK073681 from NIDDK (to YT) and by a research grant from the American Thyroid Association (to CL).

## Conflict of Interest

YT declares that he was previously (1/2015 – 6/2017) the principal investigator on a basic research project jointly funded by the Juvenile Diabetes Research Foundation and Pfizer. The current manuscript is not related to that research project. YT and CL declare that they submitted a patent application for Cepharanthine as a treatment for APS3v and T1D. YT, RO, and LF have additional patent applications that are not related to the content of this manuscript.

The remaining authors declare that the research was conducted in the absence of any commercial or financial relationships that could be construed as a potential conflict of interest.

## Publisher’s Note

All claims expressed in this article are solely those of the authors and do not necessarily represent those of their affiliated organizations, or those of the publisher, the editors and the reviewers. Any product that may be evaluated in this article, or claim that may be made by its manufacturer, is not guaranteed or endorsed by the publisher.
