# Supplementary material for: Cepharanthine Blocks Presentation of Thyroid and Islet Peptides in a Novel Humanized Autoimmune Diabetes and Thyroiditis Mouse Model
Source: Front Immunol. 2021 Dec 20;12:796552. doi: 10.3389/fimmu.2021.796552 (PMC8721038; doi:10.3389/fimmu.2021.796552)
Supplement: Supplementary file 1 [file DataSheet_1.docx]

**
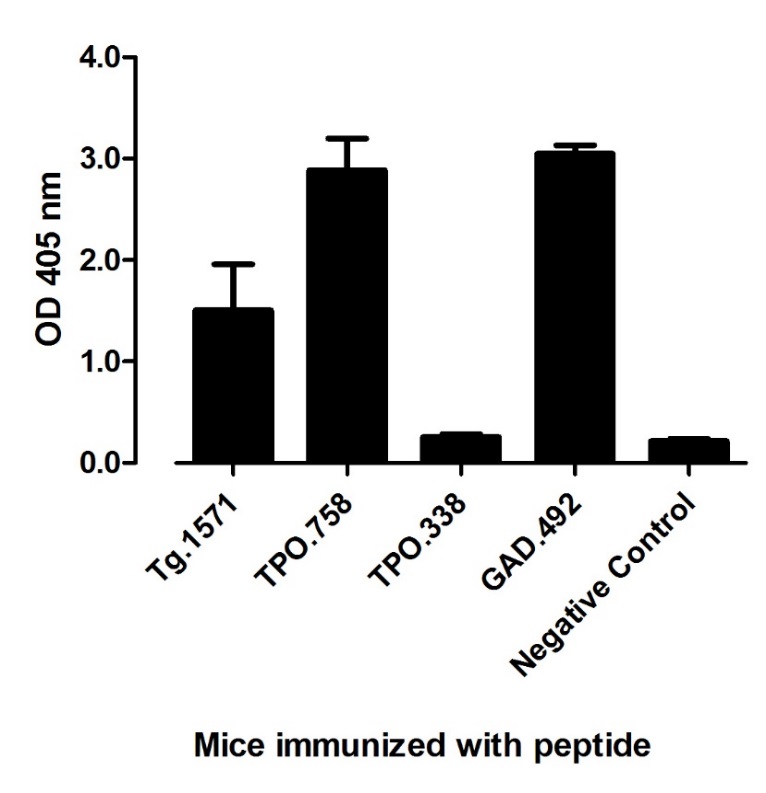
Supplemental data**

**Supplementary Figure 1**: Antibody response of NOD-DR3 mice immunized with individual peptides: Tg.1571, GAD.492, TPO.338 and TPO.758. Mice immunized with PBS + adjuvant were used as the negative control.

**Supplementary Table 1**

**Sequence of recombinant HLA-DR3 used in ELISA screening**

**DR3 Chain Sequence**

DR alpha MAISGVPV LGFFIIAVLM SAQESWAIKE EHVIIQAEFY LNPDQSGEFM FDFDGDEIFH VDMAKKETVW RLEEFGRFAS FEAQGALANI AVDKANLEIM
TKRSNYTPIT NVPPEVTVLT NSPVELREPN VLICFIDKFT PPVVNVTWLR NGKPVTTGVS ETVFLPREDH LFRKFHYLPF LPSTEDVYDC RVEHWGLDEP LLKHWEFDAP SPLPETTENE NLYFQGVDGG GLTDTLQAET DQLEDEKSAL
QTEIANLLKE KEKLEFILAA HR

DR beta MVCLRLPGGS CMAVLTVTLM VLSSPLALAG DTRPRFLEYS TSECHFFNGT ERVRYLDRYF HNQEEYVRFD SDVGEYRAVT ELGRPDAEYW NSQKDLLEQK RGRVDNYCRH NYGVGESFTV QRRVHPKVTV YPSKTQPLQH HNLLVCSVSG
 FYPGSIEVRW FRNGQEEKTG VVSTGLIHNG DWTFQTLVML ETVPRSGEVY TCQVEHPSVT SPLTVEWRAR SESAQSKMNL YFQGVDGGGR IARLEDKVKT LKAENAGLSS TAGLLREQVA QLKQKVMN
